# Salutogenesis for health promotion: tensions and future directions for physical activity/active living research

**DOI:** 10.1093/heapro/daag003

**Published:** 2026-01-30

**Authors:** Eun-Young Lee, Justin Y Jeon, John C Spence

**Affiliations:** School of Kinesiology and Health Studies, Queen’s University, 28 Division St., Kingston, ON K7L 3N6, Canada; Department of Sport Industry Studies, Yonsei University, 50 Yonsei-ro, Seoul 03722, South Korea; Department of Sport Industry Studies, Yonsei University, 50 Yonsei-ro, Seoul 03722, South Korea; Center for Exercise Medicine and Salutogenesis, ICONS, 50 Yonsei-ro, Seoul 03722, South Korea; Faculty of Kinesiology, Sport, and Recreation, University of Alberta, 3-100 University Hall, Van Vliet Complex, Edmonton, AB T6G 2H9, Canada

**Keywords:** sense of coherence, health, exercise, theory, falsification

## Abstract

A binary view of health, categorizing individuals as either healthy or diseased, has directed much of physical activity research toward evidencing its biomedical benefits. Physical activity is positioned primarily as a means of reducing risk or preventing illness with an appropriate lifestyle modification (e.g. meeting guidelines), reinforcing a pathogenic perspective. A salutogenic perspective, grounded in the ‘dis-ease–ease’ continuum, emphasizes health as a dynamic process rather than a fixed state. Within this continuum, physical activity is seen not only as preventive but also as a resource that helps individuals navigate daily stressors and move toward greater well-being. This perspective aligns with broader commitments to ‘active living,’ which extend beyond structured exercise to include how people integrate movement into everyday life. It also advances global declarations, such as the Ottawa Charter, which call for strengthening resources, enabling supportive environments, and addressing the social conditions that shape well-being. To realize this potential, physical activity research should be reframed under the broader ‘active living’ agenda through a salutogenic perspective that moves beyond risk reduction. Salutogenesis provides a compelling framework for understanding health as resource-oriented and dynamic. However, its application within physical activity research remains under-developed. Looking ahead, greater conceptual clarity is needed to explain how physical activity interacts with sense of coherence, stress management, and everyday meaning making, as well as how social and environmental factors enable or constrain these processes. Advancing salutogenesis in physical activity/active living research can move the field past binary metrics and highlight how active living fosters health-promoting, salutogenic societies.

Contribution to Health Promotion

- Health exists along a dynamic continuum that spans from ‘dis-ease’ to ‘ease,’ highlighting the fluid nature of health.
- Salutogenesis has gained increasing attention in the field of health promotion; however, its application in physical activity research remains under-developed.
- Active living, including varied forms of physical activity, is multidimensional and contextually rich, serving as a resource that supports salutary well-being.
- Actively and explicitly applying salutogenesis as a testable framework is important for advancing its theorization, bridging conceptual insights with empirical evidence, and guiding future research positioning physical activity, more broadly active living, as a vehicle for health promotion practice.

## Background

The field of physical activity research has long been dominated by a pathogenic paradigm that privileges risk reduction and disease prevention (Lee and Tremblay 2023, Gelius *et al*. 2024). This pathogenic approach led to important advances in understanding how physical activity, primarily involving physically active and less sedentary lifestyles, reduces the risk of cardiovascular disease, cancer, and premature mortality (Samitz *et al*. 2011, Ekelund *et al*. 2019). It has also played a central role in the development of evidence-based guidelines, which quantify recommended levels of physical activity for ‘optimal health benefits’ (World Health Organization 2020). Here, physical activity is commonly defined as any bodily movement produced by skeletal muscles that results in energy expenditure (Caspersen *et al*. 1985). The ‘Exercise is Medicine’ initiative (Thompson *et al*. 2020) further played a key role in elevating the importance of physical activity within clinical and public health settings. By framing exercise as a prescriptive tool to prevent and manage chronic diseases, the ‘Exercise is Medicine’ initiative contributed to policy shifts, medical education, and widespread awareness of physical activity, particularly structured, planned, goal-oriented exercise, as a key component for maintaining and improving health (Fowles *et al*. 2018, Thompson *et al*. 2020, Lindeman *et al*. 2023).

This biomedical framing has effectively highlighted the health benefits of physical activity and attracted government investment and public health interests. However, it has simultaneously constrained how physical activity is conceptualized and practiced. Specifically, a pathogenic perspective has reinforced a medicalized view that positions physical activity primarily as a prescriptive intervention. For example, much of physical activity research emphasizes the mechanisms through which specific doses of physical activity (e.g. frequency, intensity, and duration) confer health benefits or mitigate risks using epidemiologic methods. A biomedical emphasis of physical activity research has also contributed to overlooking its multidimensional benefits to broader health beyond ‘complete’ health, as denoted in the World Health Organization (WHO) (1946)’s definition of health. In a similar vein, a pathogenic perspective often reinforces such a binary view of health as either the presence or absence (‘complete’ health) of disease and positions physical activity primarily as a tool for disease prevention. The persistence of a narrow, biomedical view of health and the limited utility this framing affords to physical activity has partly contributed to the decline in federal support for physical activity research and promotion in Canada, amid a shifting health landscape that increasingly emphasizes broader dimensions of well-being (Lee and Tremblay 2023).

Critical health researchers have long critiqued the dominance of individual-focused, biomedical narratives that align with neoliberal health discourses emphasizing personal responsibility and, at times, ‘victim blaming’ (Crawford 1977, Antonovsky 1996, Quennerstedt 2008). These narratives often promote behavior change and adherence to guidelines as primary pathways to health while often overlooking broader understandings of human movement, the body, and the social, cultural, and material conditions that shape people’s capacity to move and thrive (Giles-Corti *et al*. 2015, Thompson *et al*. 2020, Lee and Tremblay 2023, Lee *et al*. 2025b). These critiques align with the concept of ‘healthism’ (Crawford 1977, 1980) where responsibility for maintaining health is individualized, and deviations from guideline adherence are implicitly moralized. The pathogenic perspective is also increasingly insufficient to capture the complex, context-dependent nature of different types and domains of physical activity embedded in social, cultural, or environmental contexts that support ‘active living’ (Makosky 1994).

**Figure 1:**
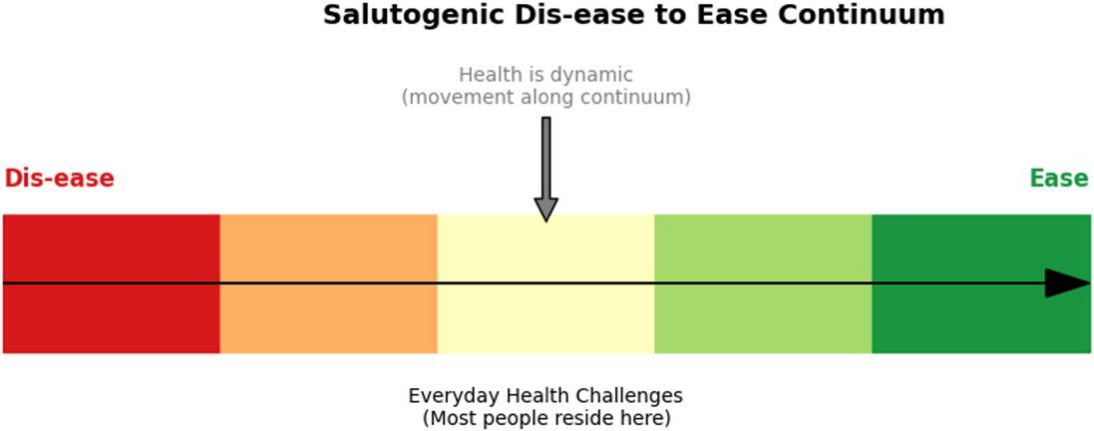
The health–dis-ease continuum conceptualized in the SMH. Note: Health is represented not as a binary state but as a dynamic process along a continuum ranging from dis-ease to ease. Individuals move along this continuum based on their ability to access and mobilize internal and external resources. Most people are situated somewhere between the extremes, constantly adapting to life’s challenges.

The Salutogenic Model of Health (SMH), first introduced by the medical sociologist Aaron Antonovsky (1979), centers the question of what makes people healthy (‘salutogenic’) rather than what makes them ill (‘pathogenic’). A salutogenic perspective is grounded in the concept of a health–dis-ease continuum (Fig. 1), emphasizing that health is not a binary state but a dynamic process in which individuals continuously move along a spectrum between ‘ease’ (well-being) and ‘dis-ease’ (distress or dysfunction), depending on their capacity to manage stressors and mobilize resources (Antonovsky 1979). It is important to note that salutogenesis does not assume health as a default but asks what makes people move toward the ‘ease’ end of the continuum (Antonovsky 1979). A growing number of researchers called for a shift toward such a salutogenic conceptualization of health as resources or as inherently desirable collateral outcomes across various fields, including health promotion (Mittelmark and Bull 2013, Bauer *et al*. 2020), education science (Lindström and Eriksson 2011), physical education (Quennerstedt 2008), sport for youth development (Super *et al*. 2021), leisure (Young *et al*. 2018), physical activities (Ericson *et al*. 2021, Boonekamp *et al*. 2022, Happ *et al*. 2024), and outdoor learning (Schreuder *et al*. 2014) and outdoor activities (Quennerstedt *et al*. 2025).

Within the salutogenic perspective, physical activity can be understood not only as a preventive tool but also a resource that can support individuals in navigating daily stressors and moving toward health and greater well-being (Quennerstedt 2008, Ericson *et al*. 2021, Boonekamp *et al*. 2022, Happ *et al*. 2024, Quennerstedt *et al*. 2025). In this work, we explore both the tensions and potential of a salutogenic perspective for physical activity. Specifically, we interrogate the prevailing reliance on pathogenic models that prioritize disease prevention and guideline adherence and highlight the need to reframe physical activity through a holistic, active living lens. We also explore the conceptual development and methodological challenges of the SMH, particularly the sense of coherence (SOC) construct, and identify areas where empirical rigor and theoretical clarity are needed. Finally, grounded in the foundational principles of the Ottawa Charter for Health Promotion (World Health Organization 1987) and subsequent declarations (World Health Organization 2005, 2018, Bull *et al*. 2010, The International Congress on Physical Activity and Public Health 2016), we call for a renewed research agenda that conceptualizes movement as a socially situated, meaning-driven, and empowering practice. We also offer actionable recommendations to position physical activity within a broader salutogenic framework of everyday active living.

## Salutogenic understanding of health

In 1946, the Constitution of the WHO defined health as ‘a state of *complete* physical, mental and social well-being and not merely the absence of disease or infirmity.’ This definition emphasizes well-being beyond just not being sick and still serves as a foundational concept of health. However, the definition faced criticism (Antonovsky 1979, Saracci 1997, Jadad and O’grady 2008, Huber *et al*. 2011, Mittelmark and Bull 2013) primarily because the term ‘complete’ sets an unrealistic and static standard, implying that health is a fixed state rather than a dynamic process (Saracci 1997, Jadad and O’grady 2008, Huber *et al*. 2011). It also overlooks those living with chronic conditions or disabilities and aged populations who still experience meaningful lives and risks over-medicalizing normal variations in human experience (Saracci 1997, Jadad and O’grady 2008, Huber *et al*. 2011). The Ottawa (World Health Organization 1987) and subsequent Bangkok (World Health Organization 2005) Charters for Health Promotion highlighted the importance of social and personal resources as well as physical capacity for health (Eriksson and Lindström 2008). [In Eriksson and Lindström’s 2008 paper, ‘A salutogenic interpretation of the Ottawa Charter (Eriksson and Lindström 2008),’ Antonovsky’s SMH is noted to influence the development of health promotion in the 1980s, but they also acknowledge that this is not explicitly stated in the Ottawa Charter. The development of public health and health promotion research positioned to the development of the salutogenesis is presented in Table 1 of the paper (Eriksson and Lindström 2008).] These declarations invite a broader understanding of health, not merely as a means to prevent disease, but as a way to enhance daily functioning, psychosocial resilience, and quality of life, especially among those who may never meet the traditional standard of ‘complete’ health (e.g. people living with chronic diseases, those with disability, or those who are aging/aged). Yet, despite these advances in conceptualizing health, the WHO’s definition remains unchanged. (The WHO recently added the following statement to its constitution: ‘The achievement of any State in the promotion and protection of health is of value to all.’)

Central to the SMH is the concept of the ‘health–dis-ease’ continuum (Antonovsky 1979), which challenges the binary framing of health (Quennerstedt 2008, Young *et al*. 2018, Mittelmark and Bauer 2022). Within a salutogenic perspective, health is viewed as a dynamic, fluid process in which individuals move along a spectrum based on their capacity to understand, manage, and find meaning in life’s stressors. Because of such fluidity in understanding health, applying a salutogenic perspective to health promotion encourages a shift from disease-oriented models toward a broader focus on what enables individuals and communities to thrive. Such a reorientation is vital for physical activity scholarship in light of growing global health challenges—climate anxiety (Hickman *et al*. 2021), digital overload (Browne *et al*. 2020), and economic and political instability (Golberstein *et al*. 2019, The U.S. Surgeon General’s Advisory 2021)—all of which reshape how health is experienced. Furthermore, mental health disorders, chronic stress, and burnout are at crisis levels, especially among youth (van Nieuwenhuizen *et al*. 2021), healthcare workers (Guille and Sen 2024), and marginalized communities (Campagne 2019, Mezza *et al*. 2024). Disease-centered models are insufficient to address these complex, context-driven stressors. In contrast, applying a salutogenic perspective toward health emphasizes building the personal and collective resources needed to adapt, cope, and thrive in challenging situations. For example, having access and opportunities to engage in activities such as leisure, outdoor, nature-based play and education (Quennerstedt 2008, Tremblay *et al*. 2015, García-Moya and Morgan 2017, McCuaig and Quennerstedt 2018, Young *et al*. 2018, Lee *et al*. 2025a, Quennerstedt *et al*. 2025), community exercise groups (Costello *et al*. 2019), and cultural sporting activities (Dubnewick *et al*. 2018) can contribute not only to physical health but also to emotional regulation, social connection, and a stronger sense of purpose; all being potential resources for movement toward the ‘ease’ end of the health continuum (Fig. 1).

A salutogenic perspective is also increasingly relevant in aging societies. While longevity dominates health metrics, extended life without autonomy or meaning is not inherently desirable. The rise in assisted dying in high-income countries reflects a deeper need to prioritize ‘healthspan’ over lifespan, supporting lives that are not just longer, but more dignified, connected, and purposeful (Pageau *et al*. 2025). Similarly, bold, public-facing claims such as ‘sitting is worse than smoking’ (Vallance *et al*. 2018) or ‘loneliness is more deadly than alcohol’ (Williams-Farrelly *et al*. 2024) highlight overlooked risks but may oversimplify complex realities of contemporary health challenges. From a salutogenic perspective, the potential harms of sedentary behavior or loneliness depend on how individuals make sense of and respond to their situations, based on the resources available to them. For example, those with strong social support, opportunities for movement, or a sense of purpose may be less affected, whereas limited resources can increase vulnerability to harm (e.g. a caregiver who sits for long hours but remains physically active otherwise, socially connected, and purposeful may be less at risk than those who are inactive, isolated, and disempowered).

## Physical activity as a salutogenic resource

Despite its longstanding and expanding interest in salutogenesis across the broader field of health promotion (Antonovsky 1996, Mittelmark and Bull 2013), its application within physical activity research remains under-developed. Only a few studies have tapped into this potential and discussed in a sufficient manner (Quennerstedt 2008, McCuaig and Quennerstedt 2018, Ericson *et al*. 2021, Happ *et al*. 2024). For example, Ericson *et al*. (2021) examined the potential of physical activity as a health resource among older adults based on empirical data, suggesting that social relations, positive energy, physical activity habits, and embodied satisfaction can support well-being.

Applying a salutogenic perspective to physical activity can reframe movement not simply as a tool to reduce disease risk, but as a resource that can strengthen SOC and enhance resilience and coping (Ericson *et al*. 2021, Boonekamp *et al*. 2022, Happ *et al*. 2024, McCurdy *et al*. 2025). This aligns with a conceptual shift from a deficit-based toward a more positive framing of movement, echoing Mittelmark and Bull (2013)’s argument on applying the SMH within health promotion research. For example, engaging in physical activity can structure daily routines, provide predictability, and support understanding of bodily and environmental cues, enhancing an individual’s agency and sense of control (Boonekamp *et al*. 2022)—all of which are relevant to comprehensibility. Furthermore, physical activity can contribute to enhancing manageability by increasing physiological resilience, cognitive and emotional coping capacity, and social connectedness, thereby increasing an individual’s capacity to handle stressors (Ericson *et al*. 2021, Happ *et al*. 2024). Physical activity also can offer intrinsic rewards, pleasure, and opportunities for purpose and social belonging, helping individuals derive meaning from their daily experiences (Ericson *et al*. 2021).

## Advancing the active living agenda: a salutogenic framing of physical activity

What is physical activity? The question is often taken for granted. Widely cited definitions such as that of Caspersen *et al.* (1985) emphasize energy expenditure and classify movement by intensity or duration. While useful for surveillance and intervention purposes, such definitions are grounded in biomedical logic and align with a pathogenic model of health, positioning movement primarily as a means to foster physical fitness and prevent illness. This framing risks overlooking the broader meanings of physical activity as an embodied, identity-forming, and joyful human experience. It can also marginalize informal, relational, or culturally specific forms of movement that may hold deep significance in people’s lives. Moreover, the binary distinction between ‘physical activity’ and ‘sedentary behavior’ tends to oversimplify lived realities, particularly for populations whose bodies and their movement are shaped by structural constraints such as aging, gender norms, labor conditions, or disability (Phoenix and Orr 2014, Fullagar 2017, Smith *et al*. 2021, Salvo *et al*. 2023).

Public health guidelines, such as the 150-minute moderate-to-vigorous activity benchmark, have become a cornerstone of physical activity research particularly in the realm of behavioral and time-use epidemiology (Pedišić *et al*. 2017, Tremblay and Ross 2020). These guidelines are often presented as universal, value-neutral goals (Tremblay *et al*. 2024), when in fact they reflect normative assumptions about time, autonomy, and capacity. Such ‘one-size-fits-all’ prescriptive targets can inadvertently undermine health promotion by positioning physical activity as necessary to maintain ‘good’ health, rather than intrinsically meaningful or pleasurable. As Crawford (1980) notes, while medicine individualizes ‘disease,’ ‘healthism’ individualizes ‘dis-ease’ where personal responsibility for maintaining health is emphasized and reenforced. To challenge this status quo, we must ask: ‘What kinds of movement are deemed health-promoting and by whom are these boundaries defined?—and what forms of activity, joy, pleasure, and movement are excluded or devalued in the process?’ Critically engaging with these questions is essential in situating physical activity research within socio-political context. Such questions can expose the limitations of strictly pathogenic approaches and highlight the need to broaden the focus toward active living, salutogenesis, and holistic well-being. Importantly, such a framing also ensures that such discussion strengthens, rather than detracts from, the relevance of research to public health and health promotion practice.

A salutogenic perspective also encourages a reimagining of physical activity as a socially constructed, contextually embedded, and experientially rich practice (Fullagar 2017)—one that promotes well-being through connection, agency, and meaning, rather than merely the avoidance of disease. Within this broader view, we must fully engage with varying domains of physical activity as vital components of the active living agenda (Makosky 1994, Bercovitz and Skinner 1996). Active living, first coined by Canadian researchers (Makosky 1994), broadened physical activity by considering movement not just as a discrete, measurable dose, but as an integral part of daily life that occurs across multiple domains including transportation, recreation and leisure, play, work, and household activities. The active living movement of the 1990s (e.g. Canada’s ParticipACTION) promoted daily activity with the explicit goal of enhancing social connection and community engagement (Bauman *et al*. 2004, Pratt *et al*. 2015). Leisure-based, voluntary activities such as hiking, as well as outdoor play and community-based sport, often emphasize intrinsic motivation, enjoyment, and psychosocial benefits over biomedical outcomes (Young *et al*. 2018, Lee *et al*. 2022). These forms of physical activity align with broader paradigms that value holistic well-being. The ongoing challenge lies in expanding physical activity scholarship to deeply explore processes, experiences, and outcomes beyond disease prevention including those related to mental, social, and existential well-being.

## Revitalizing global declarations through the salutogenic framing of physical activity

Both the Ottawa Charter for Health Promotion (World Health Organization 1987) and Bangkok Charter for Health Promotion in a Globalized World (World Health Organization 2005) emphasize empowerment, equity, and the creation of supportive environments as foundational strategies for promoting health. These principles closely align with the SMH, which focuses on enhancing individuals’ and communities’ capacity to make sense of, manage, and find meaning in their lives (Eriksson and Lindström 2008, García-Moya and Morgan 2017). Building on these two Charters (World Health Organization 1987, 2005), the 2010 Toronto Charter for Physical Activity (Bull *et al*. 2010) provided a landmark call to action for governments, organizations, and communities to prioritize physical activity within health and social policy agendas. The Toronto Charter emphasizes four strategic areas including implementation of supportive environments, integration of physical activity into multiple sectors, promotion across the life course, and investment in sustainable infrastructure, all of which resonate strongly with a salutogenic perspective. The Toronto Charter also makes an effort by situating physical activity not merely as an individual-level behavior for disease prevention but as a vehicle for fostering meaning, resilience, and social connectedness within the broader active living agenda.

Although the Toronto Charter offers important guidance for advancing physical activity promotion, its implementation and interpretation have been shaped by a persistent emphasis on individual responsibility. By prioritizing access, opportunity, and behavior change within a biomedical framework, the Charter may have inadvertently narrowed the scope of physical activity promotion and drawn critique for its limited alignment with the broader, equity-oriented principles of health promotion (Lamb Drover 2014). As a result, the Toronto Charter’s relevance has been largely overlooked in the field of physical activity research, which remains dominated by behavioral epidemiologic and disease prevention paradigms (Sallis *et al*. 2000, Lee and Tremblay 2023). This may also be partly because the field of health promotion, as well as its successor areas (e.g. population health and critical public health), continues to grapple with theoretical ambiguity over the relationship between micro-level well-being (e.g. individual agency) and macro-level social systems (e.g. structure) (Labonte *et al*. 2005). The inability to explain why seemingly individual-level interventions lead to better health in a holistic and socially contingent sense may help explain why the quantitative models of population health have come to dominate public health practice over the more aspirational principles articulated in the Charters (Labonte 1997, Robertson 1998, Labonte *et al*. 2005).

Reintegrating the Toronto Charter’s guiding values with a focus on active living and salutogenic approaches can offer a powerful opportunity to reorient physical activity promotion. This approach can shift the focus from individual behavior change and guideline adherence toward addressing the broader social, environmental, economic, and political conditions that shape movement possibilities. The Toronto Charter primarily emphasizes access and opportunity, and a salutogenic perspective considers how individuals experience, derive meaning from, and are supported in their movement (Mittelmark and Bull 2013, Ericson *et al*. 2021, Happ *et al*. 2024). Increasingly, physical activity researchers are recognizing the importance of holistic well-being, extending beyond traditional biomedical outcomes to include mental, social, emotional, and even planetary health (Reis *et al*. 2022, Lee and Tremblay 2023, Siefken and Abu-Omar 2023, Gelius *et al*. 2024, Spence *et al*. 2025 , Lee *et al*. 2025a, in press). This growing interest reflects a broader understanding that physical activity, active living more broadly, contributes not only to individual health but also to sustainable and interconnected systems of what constitutes health. A salutogenic perspective also aligns research and practice with contemporary health promotion agendas, potentially offering a more effective means than the Toronto Charter for translating its principles into action. It provides both conceptual and practical guidance for developing programs, policies, and movement spaces that enhance SOC; foster resilience; mobilize personal, social, and environmental resources; and promote dignity and inclusion.

Here, to effectively translate theory into practice, physical activity can be conceptualized as a resource that directly supports resilience, coping, and holistic well-being. Physical activity can also serve as a resource generator, facilitating access to additional resources such as social connection, mastery experiences, and environmental engagement. Furthermore, physical activity can serve as an outcome of resources reflecting the availability of enabling social, material, and policy context. Together, the health promotion Charters and a salutogenic perspective could offer a compelling multi-layered, actionable framework to reimagine physical activity not simply as a tool for disease prevention at the individual level, but as part of the active living agenda that aligns with the broader notions of salutary living—a life marked by connection, meaning, and the capacity to thrive.

## From concept to practice: recommendations for future research

A recurring challenge in the physical activity field is the inconsistent notion of the SMH as theory, model, framework, or loosely defined perspective (Eriksson and Mittelmark 2022, Mittelmark and Bauer 2022). In broad health promotion research, the SMH is often ‘parachuted in’ to shape the conceptual grounding of inquiry, without advancement in its theorization (Quennerstedt 2008, Super *et al*. 2021, Quennerstedt *et al*. 2025). Furthermore, its main construct, SOC, is often invoked without clear theoretical grounding (Eriksson 2022, Eriksson and Mittelmark 2022), and the SMH is being called for more as a rhetorical device than a robust theory that can be tested and falsified (Quennerstedt 2008, Super *et al*. 2021, Quennerstedt *et al*. 2025). This weakens the potential of applying the salutogenic perspective to challenge dominant health paradigms and instead reinforces confusion and doubt around the SMH.

The SOC, comprising comprehensibility, manageability, and meaningfulness, is widely recognized as the cornerstone of the SMH (Eriksson 2022), yet its operationalization varies widely (Eriksson and Lindström 2005). Some studies reduce it to a numeric score, while others adapt it contextually but lose fidelity to the original ‘theory.’ The dynamic interplay between comprehensibility, manageability, and meaningfulness is also often lost in translation. Furthermore, more work is needed to clarify whether SOC should be treated as a trait, a state, or a process and how it shapes interventions in physical activity contexts. We offer the following recommendations to support researchers in integrating a salutogenic perspective within physical activity research.

### Expand the definition of health and physical activity

Move beyond behavioral epidemiologic frameworks that primarily link certain modes and intensities of physical activity to binary health. A salutogenic perspective calls for a more inclusive understanding of physical activity, one that adopts the broader notion of ‘active living’ and encompasses diverse domains such as leisure, play, sport, and recreation across the lifespan. This redefinition should align with the broader notion of ‘active living’ (Makosky 1994) grounded in the principles of the Toronto Charter, acknowledging activities that promote meaning, connection, and joy, not only those aimed at reducing risk.

### Shift beyond guideline adherence

We must reframe and move beyond from the emphasis on meeting physical activity guidelines as the sole indicator of health. Instead, consider how movement that constitutes active living fosters meaningfulness, coherence, and resilience in everyday life. This may include informal, incremental, and socially embedded activities—what might be described as ‘doing stuff’ with others (Makosky 1994)—that contribute to physical, mental, social, and spiritual well-being through social connectedness. Emerging evidence highlights the health benefits associated with relatively modest increases in daily step counts, reinforcing the value of accessible, everyday movement that constitutes active living (Stens *et al*. 2023, Ding *et al*. 2025). Accordingly, there is a need to develop new tools that capture the experiential, relational, and contextual dimensions of movement including informal and embodied practices often overlooked in conventional metrics (Fullagar 2017, Stens *et al*. 2023).

### Test theoretically informed hypothesized mechanisms

Within a salutogenic orientation, several theoretically informed expectations in relation to physical activity can be articulated.

- Resource hypothesis: Participation in physical activity increases the availability of salutogenic resources, strengthening SOC and supporting movement toward the ease end of the continuum.
- Mediation hypothesis: SOC mediates the relationship between physical activity and multidimensional well-being, explaining how resources translate into health outcomes.
- Moderation hypothesis: The salutogenic effects of PA are influenced by the presence of contextual resources, including social support, accessible infrastructure, and policies that enable active living.
- Multi-level interaction: Physical activity operates across multiple levels—individual, community, and structural—with the interactions between these levels shaping the overall salutogenic impact.

These propositions can also allow researchers and practitioners to move beyond dose-based, guideline-focused approaches, situating physical activity within a framework that recognizes context, meaning, and individual–environment interactions.

### Center equity through foundational health promotion Charters

Grounding efforts to promote physical activity in the foundational principles of the Ottawa and Bangkok Charters (World Health Organization 1987, 2005) which emphasize empowerment, enabling environments, and equity. These Charters provide a critical framework for ensuring that definitions of ‘health-promoting’ active living reflect diverse cultural, social, and embodied knowledges. A salutogenic perspective aligns with this legacy by shifting the focus from individual risk reduction to capacity building and structural transformation. In practice, this means prioritizing codesigned and community-led approaches that affirm lived experiences and expand opportunities for inclusive, culturally meaningful movement that constitutes active living. It also involves addressing structural barriers and creating safe, affirming spaces for all bodies to engage in active living as part of everyday life.

As we look ahead to the future of physical activity (primarily exercise)-based programs and interventions, physical activity must be redefined and repositioned as more than a ‘health-promoting’ behavior with medical utility but the one that is ‘medicinal (Celidwen *et al*. 2023).’ It should be understood as a bodily practice in a daily active living that fosters coherence and nurtures social connection and as the one that offers pathways to factors such as dignity, joy, and belonging. To advance this broader vision, future research might explore how physical activity functions as a key resource for ‘ease’ health across multiple levels, from the individual to the community and societal domains, those that are beyond biomedical outcomes. For example, at the individual level, physical activity can enhance resilience, SOC, agency, psychological well-being, and social connectedness (Young *et al*. 2018, Ericson *et al*. 2021, Boonekamp *et al*. 2022, Happ *et al*. 2024, Lee *et al*. 2025a, McCurdy *et al*. 2025). At the community and societal levels, it can foster inclusive, friendly environments, social cohesion, and supportive infrastructure that enable active living for diverse populations (García-Moya and Morgan 2017, Super *et al*. 2021, Gao *et al*. 2025, Lee *et al*. 2025a, Quennerstedt *et al*. 2025). Research could also investigate how these individual and collective resources interact dynamically, shaping both health outcomes and opportunities for engagement and how interventions can be designed to maximize the salutogenic potential of physical activity across varied contexts.

## Integration of a salutogenic perspective into future physical activity/active living research

A binary view of health, categorizing individuals as either ‘healthy’ or ‘diseased’ has historically shaped much of physical activity research. This binary view further positions physical activity as a means of reducing risk or preventing illness (e.g. meeting or not meeting guidelines), reinforcing a pathogenic perspective. In contrast, a salutogenic perspective, grounded in the concept of a ‘dis-ease–ease continuum,’ emphasizes that health is not a fixed state but a dynamic process. Within this continuum, physical activity, active living more broadly, is understood not only as a preventive tool but also as a resource that can support individuals in navigating daily stressors and moving toward healing and greater well-being.

Framing physical activity as a resource encourages health promotion strategies that are practical, context based, and socially responsive to the socio-political and environmental conditions that shape movement opportunities. By focusing on the resources that are available to, and can be developed by, engaging in active living, physical activity interventions based on a salutogenic perspective can enhance individuals’ capacity to cope with daily stressors, build resilience, and support the SOC, thereby contributing to creating salutary living for all and, in turn, salutogenic societies (Eriksson and Lindström 2008). The salutogenic perspective also highlights the roles of socio-political and environmental resources that are closely aligned with sustained physical activity participation including, but not limited to, supportive communities, safe and accessible spaces, and opportunities for meaningful engagement. In doing so, physical activity is not only a means of reducing disease risk but also a tool that supports individuals and communities move toward greater health along the ‘dis-ease–ease’ continuum.

## Data Availability

Not applicable. This study did not generate or analyze any datasets.
